# Folate Deficiency, Hyperhomocysteinemia, Low Urinary Creatinine, and Hypomethylation of Leukocyte DNA Are Risk Factors for Arsenic-Induced Skin Lesions

**DOI:** 10.1289/ehp.11872

**Published:** 2008-09-26

**Authors:** J. Richard Pilsner, Xinhua Liu, Habibul Ahsan, Vesna Ilievski, Vesna Slavkovich, Diane Levy, Pam Factor-Litvak, Joseph H. Graziano, Mary V. Gamble

**Affiliations:** 1 Department of Environmental Health Sciences; 2 Department of Biostatistics; 3 Department of Epidemiology, Mailman School of Public Health, Columbia University, New York, New York, USA; 4 Department of Pharmacology, College of Physicians and Surgeons, Columbia University, New York, New York, USA

**Keywords:** arsenic, Bangladesh, DNA methylation, epigenetics, folate, folate deficiency, global methylation, homocysteine, hyperhomocysteinemia, skin lesions

## Abstract

**Background:**

Arsenic methylation relies on folate-dependent one-carbon metabolism and facilitates urinary As elimination. Clinical manifestations of As toxicity vary considerably among individuals and populations, and poor methylation capacity is thought to confer greater susceptibility.

**Objective:**

After determining that folate deficiency, hyperhomocysteinemia, and low urinary creatinine are associated with reduced As methylation, and that As exposure is associated with increased genomic methylation of leukocyte DNA, we asked whether these factors are associated with As-induced skin lesion risk among Bangladeshi adults.

**Methods:**

We conducted a nested case–control study of 274 cases who developed lesions 2 years after recruitment, and 274 controls matched to cases for sex, age, and water As.

**Results:**

The odds ratios and 95% confidence intervals (CIs) for development of skin lesions for participants who had low folate (< 9 nmol/L), hyperhomocysteinemia (men, > 11.4 μmol/L; women, > 10.4 μmol/L), or hypomethylated leukocyte DNA at recruitment (< median) were 1.8 (95% CI, 1.1–2.9), 1.7 (95% CI, 1.1–2.6), and 1.8 (95% CI, 1.2–2.8), respectively. Compared with the subjects in the first quartile, those in the third and fourth quartiles for urinary creatinine had a 0.4-fold decrease in the odds of skin lesions (*p* < 0.01).

**Conclusions:**

These results suggest that folate deficiency, hyperhomocysteinemia, and low urinary creatinine, each associated with decreased As methylation, are risk factors for As-induced skin lesions. The increased DNA methylation associated with As exposure previously observed, and confirmed among controls in this study, may be an adaptive change because hypomethylation of leukocyte DNA is associated with increased risk for skin lesions.

Chronic arsenic exposure is a major health concern in at least 70 countries, and an estimated 140 million people are exposed to levels of As in drinking water that exceed the World Health Organization standard of 10 μg/L (Bagchi 2007; Kinniburgh et al. 2003; Smith et al. 2000). Chronic ingestion of As through drinking water is associated with cancers of the skin, liver, lung, and bladder (National Research Council 2001), ischemic heart disease (Tseng et al. 2005), and neurologic consequences in both adults and children (Hansen et al. 2004; Wasserman et al. 2004). Premalignant skin lesions (i.e., melanosis, keratosis, and leukomelanosis) are hallmarks of chronic As ingestion by humans and, unlike internal cancers, which take decades to present, can develop within a few years of exposure (National Research Council 2001; Saha 2003). Previous studies showed strong dose–response relationships between As exposure and skin lesions (Ahsan et al. 2006b; Guha Mazumder et al. 1998). Because most As-induced basal and squamous-cell skin cancers develop from these premalignant lesions, skin lesions may be considered precursors of skin cancers (Ahsan et al. 2000). Although the mechanism by which As induces adverse health effects has not been clearly elucidated, multiple pathways have been suggested, including oxidative-dependent processes, inhibition of DNA repair, altered cellular proliferation, acquired apoptotic tolerance, and alterations in DNA methylation (Liu and Waalkes 2008).

Nutritional deficiencies are thought to confer increased risk for susceptibility to As-induced skin lesions. Case–control studies using dietary questionnaires for nutritional assessment have found that undernourishment and low intake of folate, animal protein, calcium, and fiber are associated with elevated risk for skin lesions and cancers (Hsueh et al. 1995; Mitra et al. 2004). Similarly, individuals who were < 80% of the standard body weight for their age and sex had a 1.6-fold increase in the prevalence of keratosis (Guha Mazumder et al. 1998). In Pabna, Bangladesh, betel nut use increased and higher fruit and canned goods intake decreased the risk of As-induced skin lesions (McCarty et al. 2006).

Folate plays an important role in one-carbon metabolism, the biochemical pathway that mediates the transfer of methyl groups from *S*-adenosylmethionine (SAM) to numerous substrates, including As, DNA, and guanidinoacetate (GAA). The latter constitutes the final step in creatine biosynthesis and is a major consumer of SAM (Mudd and Poole 1975). Once ingested, inorganic As (InAs) may undergo one or two biomethylation reactions to generate monomethylarsonic acid (MMA) and dimethylarsinic acid (DMA), respectively. Studies from Taiwan have indicated that people with relatively lower proportions of DMA in urine have a higher risk of skin and bladder cancers (Chen et al. 2003a, 2003b) and peripheral vascular disease (Tseng et al. 2005). We previously reported a high prevalence of folate deficiency and hyperhomocysteinemia in a rural area within Araihazar, Bangladesh (Gamble et al. 2005a); these factors were subsequently found to be associated with reduced As methylation (Gamble et al. 2005b). Furthermore, in a double-blind placebo-controlled folic acid supplementation trial, we reported that folic acid supplementation to participants with low plasma folate enhanced the methylation of As to DMA and increased urinary As excretion (Gamble et al. 2006). Moreover, this intervention significantly lowered the concentrations of As in blood, primarily by lowering blood MMA (Gamble et al. 2007). This latter finding is particularly important in light of landmark work by Styblo et al. (2000) and Petrick and colleagues (Drobna et al. 2005; Petrick et al. 2000, 2001) indicating that MMA(III) is the most toxic metabolite, both *in vitro* and *in vivo*.

Other macromolecules, such as DNA, also undergo methylation reactions that depend on one-carbon metabolism. DNA methylation has several important functions in mammals, including regulation of gene expression, preservation of chromosomal integrity, parental imprinting, and X-chromosome inactivation (Hermann et al. 2004). Two patterns of DNA methylation are found to be altered in nearly all forms of cancer. First, aberrant increases in methylation of CpG-rich islands located within promoter regions—that is, gene-specific hypermethylation—is associated with transcriptional repression; this can silence genes that otherwise function to prevent tumor growth and development. Second, a relative reduction in the overall level of methylation of non-CpG-island cytosines distributed throughout the genome (i.e., genomic DNA hypomethylation) is associated with reactivation of cellular proto-oncogenes and can lead to chromosomal instability (Robertson and Wolffe 2000). Exposure to As (Chen et al. 2004; Zhao et al. 1997) and folate deficiency (Davis and Uthus 2004; Shelnutt et al. 2004) have both been associated with alterations in DNA methylation, and these and other epigenetic marks (Ramirez et al. 2008; Zhou et al. 2008) have been proposed as mediators of As-induced carcinogenesis (Mass and Wang 1997; Pilsner et al. 2007).

We therefore hypothesized that genomic DNA methylation and/or key regulators of one-carbon metabolism would influence risk for As-induced skin lesions. We report here the results of a nested case–control study of As-exposed Bangladeshi adults that prospectively assessed the associations between plasma folate, B_12_, and homocysteine; urinary creatinine; and genomic methylation of peripheral blood leukocyte (PBL) DNA and the risk for subsequent development of As-induced pre-malignant skin lesions.

## Materials and Methods

The present study derives its participants from a large parent cohort study, the Health Effects of Arsenic Longitudinal Study (HEALS), which is an ongoing prospective cohort study in Araihazar, Bangladesh. Our data on socioeconomic status indicate that this region is not particularly poor by Bangladesh standards (Center for International Earth Science Information Network 2007). The HEALS cohort study includes > 12,000 men and women between 18 and 65 years of age recruited beginning in October 2000, and whom we continue to follow at 2-year intervals. At baseline and at 2-year follow-up visits, a comprehensive clinical examination is conducted by physicians trained to diagnose arsenicosis skin lesions. Further details on clinical examinations have been reported elsewhere (Ahsan et al. 2006a; Chen et al. 2007). Skin lesions include melanosis, leukomelanosis, and keratosis. Melanosis is characterized by the hyperpigmentation of the skin over wide body surface areas. Leukomelanosis is characterized by both hyperpigmentation and hypopigmentation of the skin over wide body surface areas. Keratosis is characterized by bilateral thickening of the skin of the palms and soles (Ahsan et al. 2006b).

Oral informed consent was obtained by our Bangladeshi field staff physicians, who read an approved assent form to the study participants. This study was approved by the Institutional Review Boards of Columbia University Medical Center and the Bangladesh Medical Research Council.

### Selection of cases and controls

Of the 11,746 participants recruited by May 2002, 9,727 participants completed the baseline physical examination, provided both urine and blood samples, and were free of skin lesions; we identified 712 prevalent cases of skin lesions and excluded them from the present study (Chen et al. 2007) to ensure that exposure assessment preceded disease onset. The present analysis includes 274 incident skin lesion cases that were diagnosed at the first 2-year follow-up between November 2002 and April 2004. We individually matched an equal number of controls to cases for sex and age (within 5 years) and frequency matched them for water As (± 100 μg/L). Of the 274 skin lesion cases, 201 had melanosis, 11 had keratosis, 3 had leukomelanosis, and the remaining 59 had both keratosis and melanosis. Plasma samples were unavailable for 25 cases; we analyzed folate and B_12_ for the remaining 248 pairs. Furthermore, an adequate volume of whole blood was available for blood As analyses only for a subset of 193 pairs.

### Analytic techniques

#### Sample collection and handling

At the baseline recruitment visit, we collected whole venous blood samples into Vacutainer tubes containing serum separators and into EDTA tubes. We collected spot urine samples in 50-mL acid-washed tubes and placed both blood and urine samples into portable coolers immediately after collection. Within 2–8 hr, we centrifuged the serum separation tubes at 3,000 × *g* for 10 min at 4°C, to separate serum from cells in our laboratory located in Dhaka. We stored all samples in Dhaka in a −20°C freezer and then shipped them in a frozen state on dry ice to Columbia University for analysis. At Columbia, whole blood and urine samples were stored at −20°C. Serum samples were stored at −80°C.

#### Water As

Water As concentrations of tube wells at each participant’s home were obtained during a survey of all wells in the study region carried out between January and May 2000 (Van Geen et al. 2003). We analyzed samples at Columbia University’s Lamont Doherty Earth Observatory by graphite furnace atomic absorption (GFAA) with a Hitachi Z-8200 system (Hitachi, Tokyo, Japan), which has a detection limit of 5 μg/L. Those samples found to have nondetectable As by GFAA we subsequently analyzed by an Axiom Single Collector high-resolution inductively coupled mass spectrometry (ICP-MS; Thermo Elemental, Erlangen, Germany), which has a detection limit of 0.1 μg/L (Cheng et al. 2004).

#### Total urinary As

We measured total urinary As concentrations by GFAA spectrometry in a graphite furnace system (AAnalyst 600; PerkinElmer, Shelton, CT) in the Columbia University Trace Metals Core Lab, as described previously (Nixon et al. 1991). Our laboratory participates in a quality control program for total urinary As coordinated by Philippe Weber at the Quebec Toxicology Center (Quebec, Canada). During the course of this study, intraclass correlation coefficients between our laboratory’s values and samples calibrated at Weber’s laboratory were 0.99, whereas the within- and between-day coefficients of variation (CVs) were 3% and 12%, respectively. We analyzed urinary creatinine using a method based on the Jaffe reaction (Slot 1965) and used it to correct for differences in urine concentration.

#### Whole blood As and As metabolites

We recently demonstrated that blood As is an excellent biomarker of exposure in Bangladesh (Hall et al. 2007). In a similar fashion, whole-blood specimens were digested according to method of Csanaky and Gregus (2003). We thawed frozen samples and mixed them with 0.1 vol 5.5% Triton X-100. After the addition of 0.1 vol 150 mM aqueous mercury chloride and incubation on ice for 1 min, samples were deproteinized with 1 vol 0.66 M ice-cold HClO_4_ and centrifuged for 10 min at 4,000 rpm. The supernatant was mixed with mobile phase, injected onto the high-performance liquid chromatography (HPLC) column, and detected by ICP-MS with dynamic reaction cell (DRC; PerkinElmer). We similarly processed calibration standards of a mixture of As metabolites. ICP-MS-DRC (PerkinElmer) coupled to HPLC separates and detects six As metabolites chromatographically separated by anion exchange using a PRP-X100 column (Hamilton, Reno, NV). The mobile phase was 10 mM ammonium nitrate/ammonium phosphate, pH 9.1. Arsenocholine (AsC), arseno-betaine, MMA, DMA, As(III), and As(V) are detectable with precision in blood samples with total As concentrations as low as 3 μg/L. We report InAs as total InAs because As(III) can oxidize to As(V) during sample transport and preparation. However, most of the InAs in blood appeared as As(III). We used two types of quality control samples. We purchased blood samples from the Institut de Sante Publique du Quebec that have known concentrations of 23 different elements, including As. We also have our own set of blood samples spiked with all five metabolites, AsC, As(III), DMA, MMA, As(V), at three different levels to cover the expected range of As in unspiked samples. We ran both sets of quality control samples in the beginning of every working day and after every 10 samples throughout the day. The average within- and between-day CVs for all metabolites were 2.9% and 5.7%, respectively, and those for whole-blood As were 4.1% and 6.6%, respectively.

#### Isolation of PBL DNA

We isolated DNA from frozen whole-blood samples using FlexiGene DNA kits (Qiagen, Valencia, CA) following the manufacturer’s protocol, except that we added an additional centrifugation step at 10,000 × *g* for 5 min immediately after protease digestion to pellet any remaining proteins or lipids, and then transferred the subsequent supernatant into a new micro-centrifuge tube containing 150 μL of isopropanol. We then isolated DNA according to manufacture’s protocol.

#### Genomic DNA methylation

We determined genomic DNA methylation by the methyl acceptance assay using the method of Balaghi and Wagner (1993). We incubated DNA with [^3^H]-SAM in the presence of SssI methylase, a prokaryotic CpG-specific methylase enzyme from *Spiroplasma*, which indiscriminately methylates all unmethylated cytosines in CpG sequences. Therefore, the ability of DNA to incorporate [^3^H]methyl groups *in vitro* is inversely related to endogenous DNA methylation. Briefly, we incubated 250 ng of DNA with 3 U SssI methylase (New England Biolabs, Beverly, MA), 3.8 μM (1.1 μCi) ^3^H-labeled SAM (GE Healthcare, Piscataway, NJ), and EDTA, dithiothreitol, and Tris- HCl (pH 8.2), which we then incubated for 1 hr at 37°C. The reaction was terminated on ice and 15 μL of the reaction mixture was applied onto Whatman DE81 filter paper (Fisher, Pittsburgh, PA). We washed the filter on a vacuum filtration apparatus three times with 5 mL 0.5 M sodium phosphate buffer (pH 8.0), followed by 2 mL each of 70% and 100% ethanol. We placed dried filters each in a vial with 5 mL scintillation fluid (Scintisafe; Fisher) and analyzed them by a Tri-Carb 2100TR Liquid Scintillation Analyzer (PerkinElmer). We processed each DNA sample in duplicate, and each processing run included samples for background (reaction mixture with all components except SssI enzyme), a hypomethylation control (HeLa cell DNA), and a quality control sample (DNA extracted from a whole-blood sample). The intraassay and interassay CVs were 1.8% and 5.3%, respectively. To quantify the amount of double-stranded DNA (dsDNA) in each reaction, we used an aliquot of the assayed DNA to determine DNA concentrations using PicoGreen dsDNA Quantitation Reagent (Invitrogen, Carlsbad, CA). We expressed all disintegrations per minute (dpm) per microgram of DNA.

#### Plasma folate and B_12_

We analyzed plasma folate and total cobalamin by radio-immunoassay (Quantaphase II; Bio-Rad Laboratories, Richmond, CA) as previously reported (Gamble et al. 2005a). The within- and between-day CVs for folate were 3% and 12%, respectively, and those for cobalamin were 3% and 12%, respectively.

#### Plasma total homocysteine concentrations

We measured plasma total homocysteine concentrations by HPLC with fluorescence detection according to the method described by Pfeiffer et al. (1999), and as previously reported (Gamble et al. 2005a, 2005b). The within- and between-day CVs for total homocysteine were 3% and 12%, respectively.

### Statistical analysis

We calculated descriptive statistics for characteristics of the study sample separately for cases and controls. We tested differences in matched case and control pairs using McNemar’s test for binary variables, Bowker’s test for categorical variables, and signed rank test for quantitative variables. We used conditional logistic regression analysis to estimate odds ratios (ORs) and 95% confidence intervals (CIs) for the effect of predictors on the development of skin lesions, with and without control for potential confounders. The main predictors included indicators for low folate (< 9 nmol/L), hyperhomocysteinemia (men, ≥ 11.4 μmol/L; women, > 10.4 μmol/L), and genomic DNA hypomethylation (less than the median of 58,934 dpm/μg DNA). We log transformed variables with skewed distributions such as blood As, water As, urinary As, and urinary creatinine to reduce the impact of extreme values in the analysis. For those variables subjected to log transformation, the ORs of developing skin lesions are for the doubling of exposure levels. We conducted all the analyses using SAS software, version 9.1.3 (SAS Institute Inc., Cary, NC).

## Results

Table 1 presents the characteristics of the study population. We found no significant differences in baseline body mass index (BMI) or sociodemographic variables such as house type and education between matched skin lesion case and control pairs. Although loosely matched on age (± 5 years) and well water As (± 100 μg/L), controls were younger and had lower water As levels than cases (*p* < 0.0001 and 0.02, respectively). Cases were more likely to report betel nut use than were controls (*p* = 0.02) and had higher concentrations of blood As (*p* <0 .0001) and urinary As adjusted for creatinine (*p* <0 .0001); however, in the latter case, this difference was driven in part by significantly higher concentrations of urinary creatinine in the controls (*p* = 0.02). Unadjusted urinary As concentrations did not significantly differ between cases and controls. Although there was no significant difference in mean plasma folate concentrations between cases and controls, using published reference values for plasma folate (Christenson et al. 1985), 69.5% of cases versus 58.4% of controls had low plasma folate (< 9 nmol/L; *p* = 0.009). Furthermore, plasma homocysteine concentrations were higher in cases than controls (*p* = 0.03). With sex-specific cut-offs derived from the Third National Health and Nutrition Examination Survey (Selhub et al. 1999), cases had a higher prevalence of hyperhomocysteinemia (men, = 11.4 μmol/L; women, > 10.4 μmol/L) than did controls (56.2% vs. 46.0%, respectively; *p* = 0.009). Mean genomic methylation of PBL DNA did not differ between cases and controls. Demographic and clinical data did not differ between the 274 pairs and the subsets of 233 and 193 pairs that we used for plasma folate and blood As analyses, respectively (data not shown).

Table 2 provides the unadjusted and adjusted ORs for skin lesions derived from conditional logistic regression analysis. Using sex-specific cutoffs for hyperhomocysteinemia, individuals with hyperhomocysteinemia were 1.7 (95% CI, 1.1–2.6) times more likely to have skin lesions compared with those without hyperhomocysteinemia after controlling for age, urinary As and creatinine, and betel nut use. The subset of 233 pairs with plasma folate and B_12_ concentrations had an estimated OR of 1.8 (95% CI, 1.1–2.9) for skin lesions comparing individuals with and without low folate nutritional status (< 9 μmol/L). B_12_ deficiency was not associated with the odds of skin lesions. Betel nut use conferred a modest 1.5-fold (95% CI, 1.0–2.2) increase in the odds in skin lesion development compared with those who did not use betel nut, after adjusting for covariates.

The interaction between hyperhomocysteinemia and folate status, although not statistically significant, suggested a possible joint effect of these variables (*p* = 0.08). To aid in interpretation, we stratified individuals into four groups to determine the combined effect of hyperhomocysteinemia and/or low folate on skin lesion development. Compared with the reference group (low homocysteine and high folate), individuals who had normal homocysteine but low folate or high folate and hyperhomocysteinemia had an OR of 2.0 (95% CI, 1.1–3.5) and 2.4 (95% CI, 1.1–5.4) for skin lesions, respectively. Moreover, individuals with both hyperhomocysteinemia and low folate at the time of enrollment had a similar OR, 2.3 (95% CI, 1.3–3.9) for skin lesions.

Individuals with hypomethylation of PBL DNA (i.e., dpm per microgram DNA > median) were 1.8 (95% CI, 1.2–2.8) times more likely to have skin lesions after adjusting for age, urinary As and creatinine, and betel nut use. Using tertiles of DNA methylation, the estimated ORs for individuals in the second and third tertiles of [^3^H]-methyl incorporation (higher dpm values, i.e., lower PBL DNA methylation) compared with the lowest tertile were 1.6 (95% CI, 1.0–2.5) and 1.5 (95% CI, 0.9–2.5) for skin lesion risk after adjusting for covariates (Table 2).

Finally, for every doubling of urinary creatinine concentrations (range, 5–376 mg/dL), the estimated adjusted OR for skin lesions was 0.7 (95% CI, 0.5–0.8). Further analyses using quartiles of urinary creatinine showed that, compared with the first quartile, individuals in the third and fourth quartile for urinary creatinine had an estimated adjusted OR of 0.4 for skin lesion development (*p* < 0.01) (Table 2).

Consistent with our previous study (Pilsner et al. 2007), in controls we observed negative correlations between dpm per microgram PBL DNA and various markers of As exposure including urinary As, urinary As per gram creatinine, and blood As (Spearman coefficients: −0.12, *p* = 0.04; −0.11, *p* = 0.07; and −0.19 *p* = 0.007, respectively), suggesting that As exposure is associated with increased genomic methylation of PBL DNA. After stratifying by folate nutritional status (< 9 nmol/L vs. ≥ 9 nmol/L) the negative association between blood As and dpm per microgram PBL DNA among controls was significant only in individuals with adequate folate nutritional status (Spearman correlation coefficient: ≥ 9 nmol/L, −0.26, *p* = 0.02, vs. < 9 nmol/L, −0.15, *p* = 0.09). These data support our previous findings indicating that As exposure is positively associated with genomic methylation of PBL DNA and, furthermore, that this association is modified by folate nutritional status (Pilsner et al. 2007). We found no associations between As exposure and methylation of PBL DNA methylation among cases.

Table 3 provides the full conditional regression model with all predictors included simultaneously. In the full model, we found that all individual predictors of skin lesion development remained significant except betel nut use, which dropped out of the model. Urinary creatinine was a significant predictor of future development of skin lesions: For every doubling of urinary creatinine, the risk of skin lesions was reduced by 60% (*p* = 0.0001). Taken together, this suggests that genomic hypomethylation of PBL DNA, hyperhomocysteinemia, low folate, age, and urinary As are all risk factors for the subsequent development of skin lesions.

## Discussion

The objectives of this study were to evaluate whether genomic methylation of PBL DNA and/or factors that influence As methylation—folate nutritional status, hyperhomocysteinemia, or urinary creatinine—influence the risk for subsequent development of As-induced skin lesions. Previous nutritional studies investigating As-induced skin lesions have relied on dietary questionnaires or have measured blood nutrient concentrations in samples drawn from prevalent skin lesion cases (Chung et al. 2006; McCarty et al. 2006; Mitra et al. 2004). To our knowledge, this is the first prospective study to investigate associations between plasma concentrations of folate and homocysteine and genomic PBL DNA methylation levels and development of As-induced skin lesions.

### One-carbon metabolism, folate and homocysteine, and As methylation and toxicity

One-carbon metabolism facilitates the transfer of one-carbon units that are ultimately used either for nucleotide biosynthesis or for the methylation of a variety of substrates, including DNA, As, and GAA. All methylation reactions generate the methylated product and *S*adenosylhomocysteine (SAH). Hydrolysis of-SAH generates homocysteine and adenosine; this reaction is reversible with equilibrium dynamics that favor SAH synthesis rather than hydrolysis. Removal of SAH can be achieved by downstream remethylation of homocysteine using methyl groups donated by 5-methyl tetrahydrofolate or betaine. In folate deficiency, concentrations of homocysteine, and consequently SAH, are elevated (Yi et al. 2000). Because SAH is a potent product inhibitor of most methyltransferase enzymes, including DNA methyltransferases (Cox et al. 1977) and As methyltransferase (De Kimpe et al. 1999), its efficient removal is critical for maintaining transmethylation reactions.

Population-based studies in Taiwan have indicated that individuals whose urine contains lower proportions of DMA and higher proportions of MMA are at a higher risk for skin lesions (Ahsan et al. 2007), skin and bladder cancers (Chen et al. 2003a, 2003b), and peripheral vascular disease (Tseng et al. 2005). Thus, factors that influence As methylation may influence risk for As-induced disease. We previously found significant associations between homocysteine and/or folate nutritional status and As methylation (Gamble et al. 2005b). In addition, in a randomized trial in Bangladeshi adults, folic acid supplementation to participants with low plasma folate enhanced As methylation and lowered blood As compared with placebo (Gamble et al. 2006, 2007), because of the shorter circulating half-life of DMA compared with other As species. Our findings suggest that both folate deficiency and hyperhomocysteinemia are risk factors for the development of skin lesions. Moreover, hyperhomocysteinemia, low plasma folate, or the combination of both each conferred a similar 2-fold increase in risk. We propose that this risk is attributable to the reduced capacity to methylate As. Although B_12_ is an essential cofactor for methionine synthase, the enzyme that catalyzes the remethylation of homocysteine to methionine, we found no increase risk for skin lesions among individuals with low B_12_ concentrations. Finally, betel nut use generates reactive oxygen species (Nair et al. 2004), which could affect folate levels through oxidative degradation. We previously found betel nut use to be negatively associated with plasma folate (Gamble et al. 2005a; Pilsner et al. 2007), so it is not surprising that betel nut use did not add to the model once we included folate status.

### As and genomic PBL DNA methylation

Genomic DNA hypomethylation commonly occurs in tumors and transformed cells and is thought to constitute an early event in some cancers (Robertson and Wolffe 2000). Genomic methylation of PBL DNA is reported to be positively associated with plasma folate concentrations (Pilsner et al. 2007; Shelnutt et al. 2004) and negatively associated with plasma homocysteine and SAH concentrations (Ingrosso et al. 2003; Yi et al. 2000). Among individuals with As-induced skin lesions, gene-specific promoter DNA hypermethylation of p53 and p16 in PBL DNA has recently been reported (Chanda et al. 2006). Previous animal studies have suggested that As induces hepatic genomic hypomethylation of DNA (Chen et al. 2004; Zhao et al. 1997).

Although PBLs are not known targets of As, As is an effective therapeutic agent in the treatment of acute promyelocytic leukemia, indicating that As distributes to PBL progenitor cells and influences their cellular function. Also, approximately 62% of PBLs are neutrophils that are relatively short-lived (< 1 week). Because these cells are rapidly dividing, they may respond more quickly to factors that influence DNA methylation than do cells that turn over more slowly.

In the present study, we found that individuals with genomic hypomethylation of PBL DNA had a 1.8-fold increase risk for skin lesions, suggesting that changes in genomic methylation of PBL DNA may serve as an early biomarker of molecular events associated with the initiation and/or progression of As-induced skin lesions. In a previous study of apparently healthy adults, we found As exposure to be positively associated with genomic methylation of PBL DNA, and that folate nutritional status modified this effect (Pilsner et al. 2007). The present study confirms both of these findings among controls, whereas we found no significant associations among skin lesion cases. We speculate that adequate folate may be permissive for an adaptive increase in genomic methylation of PBL DNA associated with As exposure, and that individuals who are similarly exposed but in whom the increase in genomic DNA methylation does not occur (or cannot be sustained) are at elevated risk for skin lesions.

The underlying mechanisms and physiologic consequences of As-induced alterations in genomic DNA methylation are unknown. As(III) (5 μM) exposure for 29 weeks has resulted in genomic DNA hypomethylation in human prostatic epithelial cells, which was accompanied by reduced DNA methyltransferase activity (Benbrahim-Tallaa et al. 2005). Chronic As exposure was also shown to cause hypermethylation of p16^INK4a^ and RASSF1A promoter regions in mouse lung tissue, implicating epigenetic alterations in tumor suppressor genes in As-induced lung carcinogenesis (Cui et al. 2006). Moreover, *in utero* As exposure was reported to induce a loss of methyl groups in CpG-rich regions in newborn mouse liver (Xie et al. 2007).

### Urinary creatinine and risk for As-induced premalignant skin lesions

Numerous studies have shown that urinary creatinine is a strong predictor of As methylation (Ahsan et al. 2007; Gamble et al. 2005b, 2006; Hall et al. 2007). The present study provided an opportunity to test the clinical relevance of this serendipitous finding. Remarkably, higher urinary creatinine concentrations were associated with a reduced risk for skin lesions; every doubling of urinary creatinine concentrations was associated with a roughly 60% reduced risk for skin lesions development.

GAA is formed in a reaction catalyzed by l-arginine:glycine amidinotransferase (AGAT). GAA is then methylated by GAA methyltransferase to generate creatine. Increases in plasma creatine concentrations, whether derived from *de novo* biosynthesis or exogenous sources such as protein in the diet, down-regulate endogenous creatine biosynthesis by pretranslational repression of AGAT expression. Creatine is nonenzymatically degraded to creatinine, which is excreted in urine at a relatively constant rate, so it is commonly used to adjust urinary As and many other metabolites for fluctuations in hydration. However, recent papers have highlighted the potential for introducing bias or confounding due to the influence of age, sex, and muscle mass on urinary creatinine (Barr et al. 2005; Gamble and Liu 2005). The present finding adds another dimension to this issue. Why urinary creatinine is so robustly associated with As methylation, and here with risk for skin lesions, is completely unknown. One possibility is that the down-regulation of endogenous creatine biosynthesis in response to ingestion of dietary creatine may spare methyl groups and thereby facilitate other transmethylation reactions, such as As methylation. However, additional studies will be required to elucidate the mechanism underlying these findings.

In conclusion, our results suggest that factors that influence folate-dependent one-carbon metabolism play important roles in the risk for future development of As-induced skin lesions, presumably due to their influence on As methylation. We found that folate deficiency, hyperhomocysteinemia, and genomic hypomethylation of PBL DNA are all independent risk factors for skin lesions. The novel finding of the protective role of urinary creatinine in the development of skin lesions warrants further investigations; we speculate this may be related to the role of one-carbon metabolism in creatine biosynthesis. We have confirmed that As exposure is associated with increased DNA methylation and that this effect is modified by folate status among controls. We propose that folate is permissive for an adaptive increase in DNA methylation and protective against hypomethylation of DNA that would otherwise increase risk for As-induced health outcomes. The findings of the present study support this hypothesis because they indicate that hypomethylation of leukocyte DNA is associated with increased risk for skin lesions. Furthermore, these findings suggest that methylation of PBL DNA may serve as a functional biomarker to identify individuals at risk for future skin lesion development. Large-scale long-term intervention studies will be required to determine if therapeutic strategies such as nutritional manipulation of one-carbon metabolism, which have the potential to increase As methylation and lower blood As, may lessen the burden of As-induced skin lesions and other adverse health outcomes.

## Figures and Tables

**Table 1:** Demographic and clinical data of subjects in the current study (274 pairs).

	Controls (*n* = 274)		Cases (*n* = 274)		
Variable	Value	Median (interquartile range)	Value	Median (interquartile range)	Difference in matched pairs (*p*-value)
Male	70.4 (193)		70.4 (193)		1.0
Smoked cigarettes	58.4 (160)		60.6 (166)		0.48
Use of betel nut (272 pairs)	48.9 (133)		58.1 (158)		0.02
House type					0.36
Thatched/other	11.0 (30)		9.1 (25)		
Corrugate	70.4 (193)		75.2 (206)		
Semi/pakka	18.6 (51)		15.7 (43)		
Education (years)					0.06
None	44.2 (121)		50.7 (139)		
1–5	27.0 (74)		28.1 (77)		
> 5	28.8 (79)		21.2 (58)		
Age (years)	44.1 ± 9.6	45 (37–51)	44.7 ± 9.7	45 (38–52)	< 0.0001
BMI (270 pairs)	19.6 ± 3.1	19.4 (17.4–21.4)	19.4 ± 2.9	18.8 (17.4–21.2)	0.39
Water As (μg/L)	142.9 ± 140.1	114 (26–208)	148 ± 140	117 (41–205)	0.02
Urinary As (μg/L)	159.8 ± 157.9	113 (55–205)	171 ± 160	121 (61–236)	0.49
Urinary As/Cr (μg/g cr)	265.5 ± 213.6	203 (116–350)	355 ± 351	261 (137–464)	< 0.0001
Urinary creatinine(mg/dl)	70.6 ± 49.1	57.3 (32.2–98.1)	62.7 ± 52.8	47.1 (27.1–84.3)	0.02
Detectable blood As (μg/L) 196 pairs	11.0 ± 6.7	9.6 (6.5–14.2)	14.3 ± 10.0	11.8 (7.3–17.6)	< 0.0001
Percent blood As nondetectable	28.5 (78)		4.7 (13)		< 0.0001
Plasma folate (nmol/L) 233 pairs	9.0 ± 5.0	7.6 (5.6–11.3)	8.6 ± 5.3	7.2 (5.3–10.2)	0.10
Plasma folate < 9 nmol/L	58.4 (136)		69.5 (162)		0.009
Plasma B_12_ (pmo/L) 230 pairs	254.7 ± 140.4	222 (158–320)	250 ± 141	216 (143–308)	0.92
Plasma B_12_ < 151 pmo/L	23.0 (53)		28.3 (65)		0.16
Plasma homocysteine(μmo/L)	12.9 ± 7.7	10.9 (8.6–14.4)	13.8 ± 10.0	11.6 (9.0–15.0)	0.03
Hyperhomocysteinemia^a^	46.0 (126)		56.2 (154)		0.008
Plasma selenium (μg/L) 196 pairs	152.8 ± 25.4	150 (135–168)	148 ± 21.2	149 (133–165)	0.13
Genomic methylation of PBL DNA (DPM /μg DNA)	59,408 ± 8,041	58,666 (54,825–62,846)	59,648 ± 8,548	59,536 (55,688–62,814)	0.58

Values are % (no.) or mean ± SD. *p*-Values were calculated based on tests for difference in matched pairs: McNemar’s test for binary variables, Bowker’s test for categorical variables and signed rank test for quantitative variables.

a Defined as ≥ 10.4 μmol/L for women and ≥ 11.4 μmol/L for men.

**Table 2:** ORs (95% CIs) for predictors of skin lesions, derived from logistic models with and without control for other factors.

Predictor	Unadjusted OR (95% CI) (*n* = 274 pairs)	Adjusted OR (95% CI) (*n* = 272 pairs)
Low B_12_ (B_12_< 151 pmol/L) a	1.4 (0.9–2.2)	1.5 (0.9–1.6)^b^
Low folate (folate < 9 μmol/L)^c^	1.7 (1.1–2.6)**	1.8 (1.1–2.9)^b^,*
Hyperhomocysterinemia	1.7 (1.1–2.4)**	1.7 (1.1–2.6)^b^,*
Folate and homocysteinemia combined^c^		
Low homocysteine/high folate	1.0	1.0^d^
Low homocysteine/low folate	1.7 (1.0–2.9)*	2.0 (1.1–3.5)*
High homocysteine/high folate	1.7 (0.8–3.4)	2.4 (1.1–5.4)*
High homocysteine/low folate	2.0 (1.2–3.3)**	2.3 (1.3–3.9)**
DNA hypomethylation (DPM > median)	1.7 (1.1–2.5)*	1.8 (1.2–2.8)^b^,**
DNA methylation by tertiles (DPM/μg DNA)		
1st tertile (30,318–56,459)	1.00	1.00^b^
2nd tertile (56,460–61,689)	1.6 (1.0–2.4)*	1.6 (1.0–2.6)*
3rd tertile (61,690–95,734)	1.4 (0.9–2.3)	1.5 (0.9–2.5)
Urinary creatinine (fold increase)	0.8 (0.7–1.0)*	0.7 (0.5–0.8)^c^,#
Urinary creatinine by quartiles		
4.5–29	1.00	1.00^d^
29.1–52.3	1.0 (0.6–1.7)	0.7 (0.4–1.3)
52.4–89.9	0.7 (0.4–1.1)	0.4 (0.2–0.8)**
90.0–376	0.6 (0.4–1.0)*	0.4 (0.2–0.7)**
Betel nut use	1.5 (1.1–2.2)*	1.5 (1.0–2.2)^e^,*

a Excluding 44 pairs with unknown plasma B_12_.

b Excluding 41 pairs with unknown plasma folate.

c Controlled for age, urinary As and creatinine, and betel nut use.

d Controlled for age, urinary As, and betel nut use.

e Controlled for age, urinary As, and urinary creatinine.

* *p* ≤ 0.05;

** *p* < 0.01;

# *p* < 0.001.

**Table 3:** Adjusted ORs derived from logistic model for all predictors of skin lesions (*n* = 231 case–control pairs).

Variable	OR (95% CI)
DNA methylation	
1st tertile	1
2nd tertile	2.2 (1.3–3.8)**
3rd tertile	1.8 (1.0–3.2)*
Low homocysteine/high folate	1
Low homocysteine/low folate	2.4 (1.3–4.5)**
High homocysteine/high folate	2.7 (1.1–6.3)*
High homocysteine/low folate	2.8 (1.5–5.2)#
Urinary creatinine (fold increase)	0.6 (0.4–0.7)#
Urinary As (fold increase)	1.4 (1.0–1.6)**
Age	1.3 (1.1–1.5)**
Betel nut use	1.3 (0.8–2.1)

* *p* < 0.05;

** *p* < 0.01;

# *p* < 0.001.
